# Methods of Controlling Invasive Fungal Infections Using CD8^+^ T Cells

**DOI:** 10.3389/fimmu.2017.01939

**Published:** 2018-01-08

**Authors:** Pappanaicken R. Kumaresan, Thiago Aparecido da Silva, Dimitrios P. Kontoyiannis

**Affiliations:** ^1^Department of Pediatrics, The University of Texas MD Anderson Cancer Center, Houston, TX, United States; ^2^Department of Infectious Diseases, The University of Texas MD Anderson Cancer Center, Houston, TX, United States

**Keywords:** fungal infection, immunotherapy, chimeric antigen receptor, D-CAR^+^ T cells, cell therapy, Sleeping Beauty, CD8^+^ T cells, adoptive T cell therapy

## Abstract

Invasive fungal infections (IFIs) cause high rates of morbidity and mortality in immunocompromised patients. Pattern-recognition receptors present on the surfaces of innate immune cells recognize fungal pathogens and activate the first line of defense against fungal infection. The second line of defense is the adaptive immune system which involves mainly CD4^+^ T cells, while CD8^+^ T cells also play a role. CD8^+^ T cell-based vaccines designed to prevent IFIs are currently being investigated in clinical trials, their use could play an especially important role in acquired immune deficiency syndrome patients. So far, none of the vaccines used to treat IFI have been approved by the FDA. Here, we review current and future antifungal immunotherapy strategies involving CD8^+^ T cells. We highlight recent advances in the use of T cells engineered using a Sleeping Beauty vector to treat IFIs. Recent clinical trials using chimeric antigen receptor (CAR) T-cell therapy to treat patients with leukemia have shown very promising results. We hypothesized that CAR T cells could also be used to control IFI. Therefore, we designed a CAR that targets β-glucan, a sugar molecule found in most of the fungal cell walls, using the extracellular domain of Dectin-1, which binds to β-glucan. Mice treated with D-CAR^+^ T cells displayed reductions in hyphal growth of *Aspergillus* compared to the untreated group. Patients suffering from IFIs due to primary immunodeficiency, secondary immunodeficiency (e.g., HIV), or hematopoietic transplant patients may benefit from bioengineered CAR T cell therapy.

## Introduction

Opportunistic invasive fungal infections (IFIs) are a major threat to the immunocompromised individual; neutropenia is a major risk factor for these infections (1, 2). Patients who require prolonged immunosuppressive therapy, for example, those who have undergone solid organ transplantation or hematopoietic stem-cell transplantation (HSCT) and those who have severe autoimmune diseases are also highly susceptible to IFIs (3–7). Other risk factors include long-term stays in an intensive care unit, the use of indwelling catheters, chemotherapy, or broad-spectrum antibiotics. The main causative agents of IFI are *Aspergillus* spp., *Candida* spp., and *Cryptococcus* spp. The incidence of IFI is increasing worldwide (2, 8, 9) (Table 1), and the worldwide crude mortality rate of invasive aspergillosis and invasive candidiasis has been estimated to be 0.4 deaths per 100,000 people. However, mortality rates associated with IFIs in immunocompromised patients are considerably higher, reaching 60–85% for invasive aspergillosis. The emergence of fungal strains that are resistant to currently available antifungal drugs such as polyenes, triazoles, and echinocandins poses a dangerous problem (10) and immune-based treatments are giving new hope to combat these deadly fungal infections (11–14).

**Table 1:** Incidence and patterns of fungal infections worldwide.

Fungal infection	Incidence per year	Reference	Main routes	Comments
Invasive aspergillosis (*Aspergillus*)	>300,000	(15)	Pulmonary	
Invasive candidiasis (*Candida*)	8–10 cases/100,000	(16, 17)	Cutaneous Oropharyngeal Gastrointestinal Genitourinary	
Mucormycosis (*Mucorales*)	1.7 cases/1000,000	(18)	Sinopulmonary Disseminated	
*Cryptococcus*	~1,000,000	(19)	Pulmonary	
*Pneumocystis* pneumonia	In the US, 9% among hospitalized HIV/acquired immune deficiency syndrome patients and 1% among solid organ transplant recipients	(20, 21)	Pulmonary	In immunocompromised patients, the mortality rate ranges from 5 to 40% in those who receive treatment. The mortality rate approaches 100% without therapy

*CNS, central nervous system*.

*Since there is a lack of epidemiological data in many countries, the world incidence rate may be overestimated*.

The host response to fungal infection depends on several factors, including the host immune status, site of infection, fungal morphotype (yeast or hyphae), cell wall complexity, and virulence traits, such as the production of fungal exotoxins (22–25). The routes of various fungal infections are listed in Table 1; the majority occurs *via* the sinopulmonary and gastrointestinal routes (22). The host immune response to fungal infection occurs in a coordinated way *via* both innate and adaptive immune cells. Innate effector cells, mainly macrophages and neutrophils, are the first line of defense against inhaled fungal spores (11, 26). As a result, most initial fungal encounters go unnoticed (27). Pattern-recognition receptors (PRRs) are a family of receptors that is composed of the C-type lectin receptors (CLRs), toll-like receptors (TLRs), Nod-like receptors, and other receptors that initiate immune responses against invading fungal pathogens. Cellular expression and signaling mechanism of the PRRs have been reviewed previously (28–30).

Most of the sugars present on the fungal cell wall are recognized by the receptors from the CLR family, underscoring the constant vigil of the host innate immune system against invading fungal pathogens (28, 31–33). CLRs recognize the various carbohydrate glycoprotein components of the fungal cell wall, such as β-glucan or α-mannan, which trigger downstream signaling cascades that are essential for inducing protective immunity against fungi (34–37). When the fungal insult cannot be quickly controlled, adaptive immune cells, mainly CD4^+^ T cells, activate other cellular responses and antibody production. Adaptive immune cells produce cytokines to activate B cells, which in turn secrete antibodies against fungal antigens and activate the release of antimicrobial peptides from endothelial cells. Recent comprehensive reviews have already detailed the mechanisms of CD4^+^ T cells and surveyed current immunotherapeutic strategies to control fungal diseases (12, 38, 39). Despite having intact innate immune systems, patients with acquired immune deficiency syndrome (AIDS) are highly susceptible to fungal infections, highlighting the importance of the adaptive immune system. When CD4^+^ T cell counts are low, as in patients with AIDS, CD8^+^ T cells have a heightened role in controlling fungal infections (40). In this review, we focus on the functional role of CD8^+^ T cells in the immune response to fungal infections. We then discuss a new method of combating fungal infections, engineering T cells with the “Sleeping Beauty” (SB) vector system.

## Current and Future Strategies to Control Fungal Infections

### Drug Therapy

Antifungal drugs have had only modest success in reducing the high mortality rates associated with IFIs. In large part, this is because diagnosis of fungal infection and identification of the responsible organism is often delayed, leading to a delay in the administration of directed antifungal therapy. The use of available antifungal drugs is also restricted by their route of administration, spectrum of activity, and bioavailability in target tissues such as the brain (41). Additional issues include toxicity, undesirable drug interactions, and drug resistance. Use of the triazoles, for example, is limited by their interactions with statins, corticosteroids, and other drugs (42).

Despite tremendous improvements in the response rates of aspergillosis to modern antifungal agents, fatality rates of 40% are common in contemporary real life cohorts of unselected patients with leukemia and transplant recipients (43). The high rate of mortality following *A. fumigatus* infection is a result of the suboptimal diagnostic tools available, leading to late diagnosis. Other factors include rising *Aspergillus* resistance, and even more importantly, the relative ineffectiveness of existing antifungal drugs against established *Aspergillus* infections (44).

The development of effective and safe immune enhancement therapies is a major unmet need. Some patients with candidiasis struggle with poor outcomes, although this is less common in the era of widespread azole prophylaxis given to high-risk patients. Randomized controlled studies typically exclude high-risk immunosuppressed patients by use of their inclusion criteria (45).

### Immunotherapy

#### Innate Immune Cells

Immunotherapy, which comprises cell-based therapies, such as the adoptive transfer of T cells, dendritic cells (DCs), or neutrophils, and other humoral approaches, such as antibodies and recombinant pentraxins, is a viable option for control of IFIs. Among immunocompetent individuals, the innate immunity efficiently prevents and clears IFIs (26). Alveolar macrophages are the first line of fungal defense; they recognize, phagocytize, and destroy fungal spores (46). Neutrophils also play a key role in killing fungal hyphae. They eliminate fungal hyphae by inducing an oxidative burst and by forming neutrophil extracellular traps (NETs) (47). Neutrophils utilize NETs to trap the invading pathogens by releasing chromatin fibers to form a meshwork adorned with cytoplasmic granules containing the antimicrobial enzymes myeloperoxidase, cathepsin G and neutrophil elastase that destroy trapped pathogens. The whole process is called NETosis (48).

To date, immunotherapeutic strategies to combat IFIs have primarily focused on augmenting the number of granulocytes, since these cells are known to have fungicidal activity. Granulocyte-focused immunotherapies include granulocyte transfusions (49), infusion of growth factors [granulocyte colony-stimulating factor (G-CSF), or granulocyte macrophage colony-stimulating factor (GM-CSF)] to increase granulocyte numbers (50), and the administration of cytokines such as interferon (IFN)-γ (51) and/or interleukin (IL)-15, the latter of which promotes the production of IL-8 (52), to augment phagocytic and cytotoxic function. However, the reconstitution of granulocytes is hampered by an inability to numerically expand large numbers of cells *ex vivo*. Moreover, after infusion, reconstituted granulocytes exhibit poor persistence owing to increased apoptosis, weak potency, and a propensity to become trapped in the pulmonary vasculature (53).

#### Natural Killer (NK) Cells

Natural killer cells are another type of innate immune cell reported to be involved in controlling fungal infections. NK cells make up from 5 to 15% of the peripheral blood mononuclear cells (PBMCs) of healthy individuals; the NK cell population is made up of CD56^+^ and CD3^−^ cells (54). NK cells are activated when signals from activating receptors outweigh signals from inhibitory receptors, leading to cytotoxicity directed against tumor cells and virus-infected cells. NK cells also recognize infectious fungal pathogens, including *A*. *fumigatus, C. albicans, C. neoformans*, and *Mucorales* species (31, 55–58). Recently, CD56 has been identified as a PRR that can bind directly to both germ tubes and hyphae of *Aspergillus fumigatus* (59). Upon recognition, NK cells either induce lysis of these pathogens by secreting perforin and granulysin or trigger activation of other immune cells by releasing IFN-γ (60). Fungal pathogen-specific NK cell receptors and their mechanism of action has been reviewed (61, 62).

#### Dendritic Cells

Dendritic cells are professional antigen presenting cells (APCs) that can recognize and phagocytize fungal conidia and hyphae through PRRs and degrade them by fusing with lysosome vesicles (63). PRRs activate DCs to secrete cytokines, such as IL-12, IL-6, IL-4, and IL-1β that induce T-cell differentiation in the lymph nodes. During *A. fumigatus* infection, pulmonary DCs secrete IL-12 upon exposure to conidia, while IL-4 and IL-10 are secreted after exposure to hyphae. Therefore, IL-12 signaling generates a T helper (Th) 1 cell response, while IL-4 and IL-10 signaling generates a Th2 response. DCs also secrete tumor necrosis factor (TNF)-α and the chemokine CXCL8 which recruit neutrophils to the infection site (64).

In conventional DCs, β*-*glucan-induced Dectin-1-mediated signaling promotes secretion of the cytokines IL-2 and IL-23. The release of IL-23 induces Th17 differentiation but it is tightly regulated by IL-2 (65, 66). These data suggest that DCs direct naïve T cells to mature into functional T-cell sub types by secreting specific cytokines in the microenvironment based upon stimuli received by PRRs from different forms of fungi.

#### CD4^+^ T Cells

Even though DCs help to reduce the fungal burden to some extent through fusion with lysosome vesicles, the major function of DCs is to present fungal antigens to naive T-cells. DCs present processed antigens *via* major histocompatibility complex (MHC) class I or class II molecules and interact with naive T cells through formation of an immunological synapse. T cells are broadly classified into helper CD4^+^ T cells and cytotoxic CD8^+^ T cells. In fungal infections, both CD4^+^ and CD8^+^ T cells participate in the elimination of fungal pathogens (67, 68). On the basis of their function and cytokine secretion profile, CD4^+^ T cells are classified into several subsets: Th1, Th2, Th9, Th17, Th22, regulatory T cells, and follicular helper T cells. The activity of CD4^+^ T cells against fungal infection in immunocompetent individuals has been very well characterized. The most important CD4^+^ T cells in the antifungal immune response are the Th1 and Th17 helper T cells. After priming by DCs, CD4^+^ T cells differentiate into Th1 and Th17 helper T cells. Th1 helper T cells secrete the cytokines IFN-γ and TNF-α which activate innate immune cells, such as neutrophils, macrophages, DCs, and inflammatory monocytes, to fight against invading fungi and bacteria (12, 27). The cytokines secreted by Th1 cells also activate B cells, leading to the secretion of antigen-specific antibodies against fungi. IL-17 secreted by Th17 cells controls fungal infection by mobilizing neutrophils and protecting mucosal body sites by inducing epithelial cells to secrete defensin (69). IL-17 deficiency has been shown to enhance susceptibility to *Candida albicans* infections at mucosal sites (70).

#### CD8^+^ T Cells

**Figure 1:**
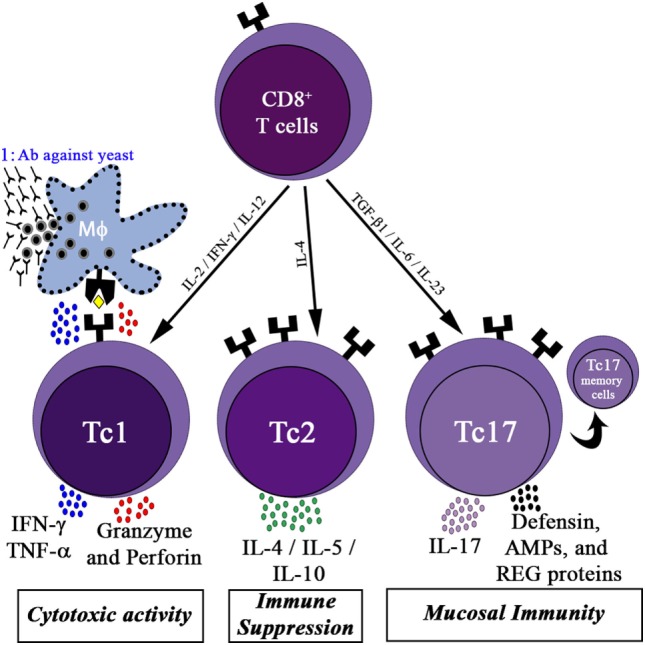
CD8^+^ T cells activity in the immune response. Differentiation of CD8^+^ T cells into three functional subsets: the cytotoxic cells (Tc1) cells, producing high levels of interferon (IFN)-γ, tumor necrosis factor (TNF)-α, granzyme, and perforin, which contribute to the killing of yeast infected host cells; Tc1 kills fungal infected macrophages and allows the participation of humoral immunity (marked as 1); Tc2 cells, release high amounts of interleukin (IL)-4 and IL-10, promoting immune suppression; Tc17 cells secrete IL-17, which activates mucosal immunity by inducing epithelial cells to secrete defensin, antimicrobial peptides (AMPs), and regenerating proteins (REG). Some of the activated Tc17 cells may differentiate into memory Tc17 cells.

Like CD4^+^ T cells, CD8^+^ T cells also have sub types, namely Tc1, Tc2, and Tc17 (Figure 1). APCs, mainly DCs, cross-present fungal antigens to CD8^+^ T cells. CD8^+^ T cells can be primed to recognize fungi by utilizing a “cross-presentation” and “cross-priming” approach, in which exogenous or fungal antigens are presented on MHC-I molecules (71). DCs internalize exogenous fungal products by CLRs and scavenger receptors for processing and presenting to MHC-I, and this process is called cross-presentation. Along with cross-presentation, some of the CLRs, for example, Dectin-1 activates DCs *via* Syk kinase signaling to produce IL-12, which favors Tc1 differentiation (72). Curdlan has been demonstrated to stimulate the Dectin-1-syk-CARD pathway, producing IL-23 to boost the differentiation of Th17 cells (73).

Upon recognition of fungal peptides presented by APCs, CD8^+^ T cells differentiate into Tc1 cells and Tc17 cells (CD8^+^ T cells that secrete IL-17A), depending on the cytokines present in the environment. Several reports highlighted the role of Tc1 and Tc17 cells in protecting humans from fungal infection (74, 75). Tc1 cells act indirectly by secreting cytokines such as IFN-γ, TNF-α, and GM-CSF to activate innate immune cells such as neutrophils and macrophages involved in antifungal defense. Furthermore, Tc1 cells directly kill unresponsive fungal-infected macrophages by secreting cytotoxic factors such as perforin, granulysin, and granzyme K (76). DCs uptake fungal breakdown products from apoptotic macrophages by endocytosis to cross prime CD8^+^ Tc1 cells. CD8^+^ Tc17 cells, like CD4^+^ Th17 cells, secrete IL-17A cytokines to activate epithelial cells (mucosal immunity) to secrete antimicrobial products such as defensin to fight against fungal infections (Figure 1).

The Tc1 and Tc17 subtypes can be divided into effector T cells and effector memory T cells on the basis of their expression of surface receptors. The cell-surface markers used for phenotypical characterization of Tc1 and Tc17 cells are shown in Table 2. Tc1 cells that express C–X–C motif chemokine receptor 3 migrate to the lungs during pulmonary infections, such as pneumocystis (77). Tc17 cells have increased levels of effector memory phenotype markers on the cell surface (CD62L^lo^ and CD27^it/lo^) as compared with Tc1 cells, suggesting that Tc17 cells may play a role in preserving long-term antifungal immunity in the host. Cytokines secreted by CD8^+^ Tc1 and Tc17 cells boost the innate immune system as well as the mucosal immune system to give protection from IFI (Figure 1).

**Table 2:** Phenotypic characterization of CD8^+^ Tc1 and Tc17 cell subtypes.

Cell-surface markers/cytokines	Expression in Tc1	Expression in Tc17	Transcription factors/secretory granules	Expression in Tc1	Expression in Tc17
CCR6	N.I.	High	TCF-1	High	High
CXCR3	High	Low	T-bet	Low/High	Low
CD62L	Low/high	Low	Eomes	High	
CD27	High	Low/intermediate	Ror (γ)t	Low	High
KLRG-1	Low	Low			
IFN-γ	+	−	Perforin	+	−
TNF-α	+	−	Granulysin	+	−
IL-2	+	−			
GM-CSF	+	−			
IL-17	−	+			

*CCR6, C–C motif chemokine receptor 6; CXCR3, C–X–C motif chemokine receptor 3; CD62L, cluster of differentiation 62L;CD27, cluster of differentiation 27; KLRG-1, co-inhibitory receptor killer-cell lectin-like receptor G1; IFN, interferon; TNF, tumor necrosis factor; IL-2, interleukin-2; GM-CSF, Granulocyte macrophage colony-stimulating factor; IL-17, interleukin-17; TCF-1, T cell factor 1; T-bet, T-box transcription factor; ROR-γt, RAR-related orphan receptor gamma; N.I., no information*.

CD8^+^ T cells share many cellular functional mechanisms with NK cells, such as releasing cytolytic granules and cytokines. As indicated above, in the absence of CD4^+^ T cells, CD8^+^ T cells also play a major role in controlling fungal infection. Immunotherapy could take advantage of several properties of CD8^+^ T cells, they can kill pathogen infected cells, be propagated in large numbers *ex vivo*, be genetically modified to recognize particular antigens, and contribute to immunologic memory.

## The Role of CD8^+^ T Cells in the Antifungal Immune Response

The immune response to fungi elicited by CD8^+^ T cells can broadly classified into two processes: (1) T-cell receptor (TCR) mediated and (2) TLR and scavenger receptor mediated.

### TCR-Mediated CD8^+^ T Cell Activation

Vaccines are a promising avenue for the treatment and prevention of IFIs (78–84) mediated through TCR receptors. The vaccine candidates developed against fungal antigens typically activate CD4^+^ T cells and Th17 cells. Several highly immunogenic and protective vaccine formulations for candidiasis are currently undergoing clinical trials (84, 85). It is worth noting that vaccination against fungi has mainly focused on yeast pathogens, such as *Candida* spp. and *Cryptococcus* spp. (80), and endemic mycoses that infect immunocompetent individuals, such as *Coccidioides* spp. (86), *Blastomyces* spp., and *Histoplasma* spp. It remains to be seen whether similar strategies will be as effective against opportunistic fungi, such as *Aspergillus*. In preclinical studies, vaccination using both crude and recombinant *Aspergillus* antigens improved the survival of immunocompromised mice following inhalation and intravenous administration of *Aspergillus fumigatus* (87).

Direct killing by CD8^+^ T cells has not been widely explored in the development of an immunotherapy against fungi, even though studies demonstrated the essential role of the CD8^+^ T-cell response in controlling fungal infections after vaccination (74, 75, 88–92) (Table 3). However, the presence of *Aspergillus*-specific CD8^+^ T cells has been shown in both mice and humans (93–96). Moreover, *Mucorales* (97) and *Fusarium*-specific T-cells (98) were reported in hematologic patients with IFI. Type I CD8^+^ T-cells (Tc1) were shown to provide protection against pneumocystis in mice (99). Preclinical studies demonstrated that the direct effect of CD8^+^ T cell-mediated cytotoxic activity and TNF-α and IFN-γ production were necessary to clear infected macrophages containing *H. capsulatum* (76), and provided full protection against coccidioidomycosis (88, 89). The activation of CD8^+^ T cells also contributed a protective response during *Cryptococcus neoformans* infection; involvement of Type 1 CD8^+^ T (Tc1) cells was triggered through immunization with the cytosolic proteins of the pathogens (90). Moreover, CD8^+^ T-cells secrete IL-17A to give protection against lethal fungal diseases, such as *Blasotomyces dermatitidis* and *Histoplasma capsulatum*, by supporting neutrophil activity (74) (Figure 1).

**Table 3:** Fungal vaccine candidates and their CD8^+^ T-cell mechanisms of action.

Fungal infection	Candidate	CD8^+^ T cell responses	Model	Reference
Aspergillosis	Recombinant fungal antigens Pep1p, Gel1p, and Crh1p	Cytotoxic activity	Murine	(100)
	Live *A. fumigatus* conidia or *A. fumigatus* cell wall glucanase Crf1p	Cytotoxic activity	Murine	(101)

Blastomycosis	Attenuated mutant lacking BAD1	Tc17 cells	Murine	(74, 91)
	Attenuated mutant lacking WI-1 adhesin	TNF-α, IFN-γ, and GM-CSF production; CD8^+^ T cell memory	Murine	(75, 92)

Coccidioidomycosis	Arthroconidia of the 95–291 strain	Cytotoxic activity; TNF-α production	Murine	(88)
	Live spores of the Δcts2/ard1/cts3 strain	IFN-γ production	Murine	(89)

Candidiasis	Candidal adhesin (rAls3p-N) plus aluminum hydroxide adjuvant	Cytotoxic activity	Murine	(102)
	*Candida dubliniensis* mannan–human serum albumin conjugate	Upregulation	Rabbit	(103)
	Cytosolic antigens entrapped in liposomes	Upregulation	Murine	(104)

Paracoccidioidomycosis	N.I.	N.I.	N.I.	N.I.

Cryptococcosis	Cytosolic proteins	Tc1 cells	Murine	(90)

*TNF, tumor necrosis factor; IFN, interferon; GM-CSF, granulocyte macrophage colony-stimulating factor; N.I., no information*.

However, there are limitations for vaccine therapy. Currently, no FDA-approved vaccines are available to prevent the major opportunistic fungal infections, specifically candidiasis, aspergillosis, and cryptococcosis. Several reasons underlie the paucity of viable candidates. First, these infections are relatively uncommon, compared to viral and bacterial infections and typically occur in severely ill patients. Thus, finding sizable niche patient population who can benefit from a cost-effective vaccine strategy is difficult and not an area of priority for development by the pharmaceutical industry. Second, high-risk patients have pleiotropic and ever-evolving defects in both innate and adaptive immunity. As responses to fungi depend on both arms of the immune response, and because such responses are complex, depending on the site of infection (mucosal vs systemic infection) and the type of fungus (e.g., *Candida* or *Cryptococcus* vs a mold), much more groundwork needs to be done to decipher the key elements of a successful vaccine. In addition, there are questions regarding the efficacy and feasibility of using a vaccine in immunocompromised patients, since they are incapable of mounting a complete immune response (105).

### TLR-Mediated CD8^+^ T Cell Activation

Toll-like receptors of the innate immune system play a major role in recognizing fungal cell wall carbohydrates, cell wall breakdown products, RNA, and DNA (13, 106–108) and thereby activate immune cells. One possible mechanism TLRs use to augment T-cell activation is when DCs activate fungal-specific CD8^+^ T cells by cross-presenting fungal antigens. TLR3 plays a crucial role in this process by sensing fungal RNA derived from necrotic cells and activating CD8^+^ memory T cells along with DCs. Indeed, TLR3^−/−^ mice are more susceptible to *Aspergillus* infection than are control mice (101), and people with mutations in key TLR3 and TLR4 signaling components are susceptible to various fungal infections (109–111).

### T-Cell Activation Mediated by Scavenger Receptors and Other Receptors

The scavenger receptor proteins are a highly heterogeneous set of proteins expressed on the cell surface that are involved in the uptake of modified low-density lipoproteins and a variety of microbes. One of the scavenger receptors, CD5, has been shown to bind β-glucan, a fungal cell wall sugar moiety, as well as many strains of yeast cells (112). CD5 is expressed on T cells and a small subset of mature B cells, where it associates with antigen receptors. Upon stimulation with zymosan (a protein-sugar moiety derived from the yeast cell wall), a CD5-transfected cell line produces IL-8, suggesting that CD5 has a pro-inflammatory role in fungal infection (112).

Besides TLRs, T cells have other receptors such as CD23 and CD56 for direct recognition of fungal antigens. CD23 is an inducible low-affinity receptor for immunoglobulin (Ig)E (113). It can recognize both β-glucan and α-mannan sugar moieties and thereby targets both yeast and hyphae forms of *Candida* (114), and upon activation, it upregulates nitric oxide production to destroy invading *Candida*. C-Jun N-terminal kinases (JNK1) activation suppresses the expression of CD23, which increases susceptibility of fungal infection. This was verified in JNK1 KO mice which showed resistance to *Candida* infection when compared to control mice (114). CD56 is a NK cell receptor that has been shown to bind to *Aspergillus* hyphae in a concentration-dependent manner. Blocking of CD56 reduced fungal-mediated NK cell activation (59). Activated T-cells expresses high levels of CD56 and its expression level directly correlates with T-cell effector functions (115). However, additional studies are warranted to verify that the CD56 mediated CD8^+^ T cells are activated during fungal infection.

## Adoptive T-Cell Therapy

Over the years, several immunotherapies have been used to treat fungal infection. One such immunotherapy, adoptive T-cell therapy (ACT), is a promising therapeutic strategy not only for cancer but also for treating viral and fungal infections (38, 116–118). ACT involves the isolation and *ex vivo* expansion of autologous T cells in an antigen-specific manner; these expanded T cells are later infused into the patient. ACT has been shown to be effective in controlling viral infections, such as cytomegalovirus (119) and fungal infections, such as *Aspergillus* in HSCT patients (120). Immunocompromised patients, especially patients undergoing allogeneic HSCT, are highly susceptible to IFIs (7). The mortality rate from IFI in this patient population remains unacceptably high, partly due to the long-lasting immunosuppression in patients after HSCT (3, 121). Most IFIs in these patients occur after engraftment of the innate immune system, which suggests that adaptive cellular immunity plays a major role in controlling IFIs. In fact, adoptive transfer of CD4^+^ Th1 cells elicited significant protection against invasive aspergillosis in haploidentical HSCT settings (120). These findings have generated a growing interest in restoring adaptive immunity against fungal pathogens by infusing donor-derived antifungal T cells and in various *ex vivo* methods of propagating clinical-grade *Aspergillus*-specific T cells (38, 122). Recently, the FDA-approved chimeric antigen receptor (CAR) T-cell therapy to treat B-cell malignancies. CAR T cell technology can be applied to redirect T cell specificity to target fungal pathogens.

Three approaches are used to redirect T-cell specificity against a particular antigen (123).

**Figure 2:**
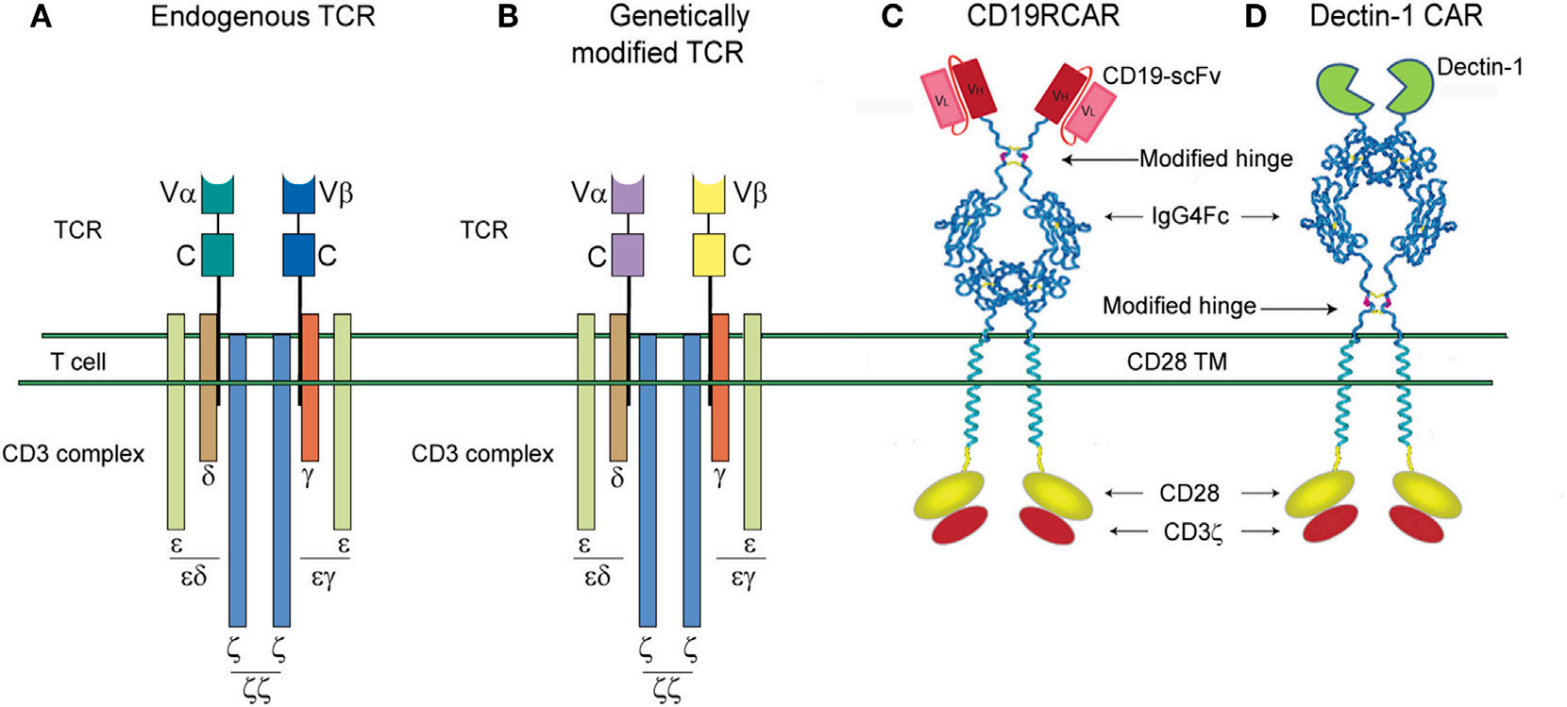
Schematic representations of the T-cell receptor (TCR) complex and second-generation single-chain variable region (scFv)-chimeric antigen receptor (CAR) and D-CAR. **(A)** Unmodified endogenous TCR complex and **(B)** genetically modified TCR complex. The α and β chains are highlighted in different colors. **(C)** The CD19R-CAR derived from a scFv region of a CD19 antigen-specific mouse monoclonal antibody and **(D)** the D-CAR^+^ derived from an extracellular domain of the Dectin-1 receptor. CD19R-CAR and D-CARs shown here have the same signaling domains, derived from costimulatory molecules, such as CD28 and CD3-ζ.

1. Gene modification with antigen-specific TCRs in which the α and β chains of the TCR are cloned from tumor-associated antigen-specific T-cell clones (124, 125).
2. Gene modification using natural receptors other than TCRs, such as the Dectin-1 receptor (126).
3. Introduction of a CAR that recognizes tumor-associated antigens through a single-chain variable region (scFv) derived from the corresponding monoclonal antibody (127). Currently, there are no reports in the literature of scFv-derived CAR T-cells targeting fungal antigens. Therefore, in the current review, it was added for comparison analysis with D-CAR^+^ T cells (Figure 2).

### Gene Modification Using Pathogen-Specific TCRs

T-cell receptors are found on the surface of T cells as heterodimers of α and β chains and they recognize antigens presented by the MHC receptors of the APCs. For ACT, genes of tumor antigen-specific TCRs are isolated from patients and engineered into T cells using a viral or non-viral-based vector system (128, 129). These T cells are expanded *ex vivo* to generate large numbers for infusion. Improvements in vector design have recently increased the efficiency of this approach, and the avidity of the TCR has been improved by substituting amino acids in its complementarity determining region and introducing cysteines to form disulfide bonds, thereby preventing α- and β-chain mispairing. TCR-mediated T-cell responses to fungal antigens have been documented in both *in vitro* and *in vivo* studies (130, 131).

In colon cancer studies, TCR-driven ACT was effective in reducing tumor volume (132), but a high incidence of toxicity was reported, especially when high-avidity TCRs were used (133). Moreover, TCR-specific therapy is MHC restricted; if tumor cells lose antigen expression by downregulating MHC, they can evade the T cell attack (134). Hence, TCR-specific T-cell recognition is restricted to a single type of MHC molecule that presents the targeted antigen (135). In order to circumvent this problem, CAR-based therapy was developed. With CAR-based T-cell therapy, the tumor recognition of the CAR is not dependent on MHC (136).

### Engineered CAR T-Cell Therapy

Engineered T-cell therapy involving the introduction of a CAR, which recognizes tumor-associated antigens through its scFv, is derived from the corresponding monoclonal antibody. CAR-based therapy involves the genetically engineered fusion of a variable light chain and a variable heavy chain that are specific for a cell-surface antigen and are tailored to produce an activating signal to host immune cells upon antigen engagement (137). CAR-based T-cell therapy has several key advantages. First, a CAR-based approach can be used in all tumor conditions expressing the antigen and is not MHC restricted. Second, tumor cells have no protection against CAR-based immunoediting. Finally, a varied range of tumor antigens can be targeted using this system, including glycoproteins and lipids (138, 139).

The CAR has been structurally refined over three generations of development. CAR’s structure consists of four elements: an antigen-targeting domain, an extracellular linker/spacer, a membrane-spanning (transmembrane) domain, and an intracellular signaling domain. The antigen-specific domain is usually derived from the scFv of the monoclonal antibody targeting the antigen. The linker makes the CAR flexible so that it can reach the antigen. A mutated IgG derived Fc sequence incapable of activating innate immune cells is commonly used because of its stability in expressing the CAR on the cell surface (140). In first-generation CARs, the transmembrane domain used CD4 or CD8, while CD28 was used in second-generation CARs (141). The intracellular signaling domain of second-generation CARs used CD3-ζ along with the costimulatory signaling domain CD28 (142). Tumor clearance and persistence is better in second- and third-generation CAR^+^ T cells than in the first generation (140, 141, 143, 144).

### SB, a Non-Viral-Based Vector

Several vector systems have been used to introduce the CAR transgene into T cells. Of these, mammalian transposon/transposase-based vectors produce the most robust integration, have low immunogenicity, and allow for easy manipulation of plasmids. Multiple vectors have been studied in mammalian systems, including the SB transposon (derived from the fish, *Tanichthys albonubes*), the PiggyBac element (from the moth, *Trichoplusia ni)*, Frog Prince (from the frog, *Rana pipiens*), Himar1 (from the horn fly, *Haematobia irritans*), Tol2 (from the fish, *Oryzias latipes*), and Passport (from the flatfish, *Pleuronectes platessa)* (145, 146).

Among all of the elements with activity in mammalian cells, the SB transposon is one of the most widely studied for use in gene transfer (147, 148). The SB transposase was derived by combining inactive transposase sequences from the genome of salmonid fish and then reversing the termination codon to activate transposase activity. A typical SB vector consists of 230-base pair (bp) regions containing long inverted and direct repeats (IR/DR) flanking the target gene sequence. These IR/DR sites bind with SB transposase to transfer the target gene to the host genome. In addition to the IR/DR sites, SB transposase also contains a DNA-recognition site, a nuclear localization signal, and a catalytic domain. Gene transfer using the SB transposon/transposase involves a cut-and-paste mechanism. The SB transposase protein is translated and accumulated in the cytoplasm, which is then imported into the nucleus using the nuclear localization signal. The SB transposase protein binds to the IR/DR sequence of the transposon, causing DNA breaks around the gene of interest.

The integration site of the gene cut from the SB transposon in the T cell genome depends on the presence of a TA dinucleotide site, DNA flexibility, and proximity of the donor and receiver (local hopping). More than 25% of integrations occur within a 200-bp region between the donor and receiver sites of the gene, and more than 75% of integrations are located in a single chromosome. CD19R-CAR T cells were developed using the SB vector and successfully used in clinical trials to treat acute myeloid leukemia and chronic lymphocytic leukemia (127, 149).

### Dectin-1 CAR T-Cells to Target β-Glucan-Expressing Fungi

We modified this prototypical CAR to recognize carbohydrates by utilizing the pattern-recognition properties of Dectin-1 (126, 150, 151). It is specific for β-glucan, a glucose polymer consisting of β-1, 3-glucan and β-1, 6-glucan that is expressed on the cell wall of all known fungi (152–155). We hypothesized that the extracellular portion of Dectin-1 could be adapted as the specificity domain for a CAR (D-CAR) on T cells to redirect their specificity to β-glucan expressing fungi such as *Aspergillus*. Using the extracellular domain of Dectin-1, we engineered a CAR with specificity for the fungal cell wall sugar moiety β-glucan. This CAR was fused in frame to a modified human IgG4 hinge/Fc stalk (156), CD28 transmembrane domain, and a combination of CD28 and CD3ζ intracellular domains. This design is similar to that of our second-generation CD19-specific CAR (designated CD19RCD28), which is currently being employed in clinical trials to treat B-cell leukemia (157) (Figure 2).

**Figure 3:**
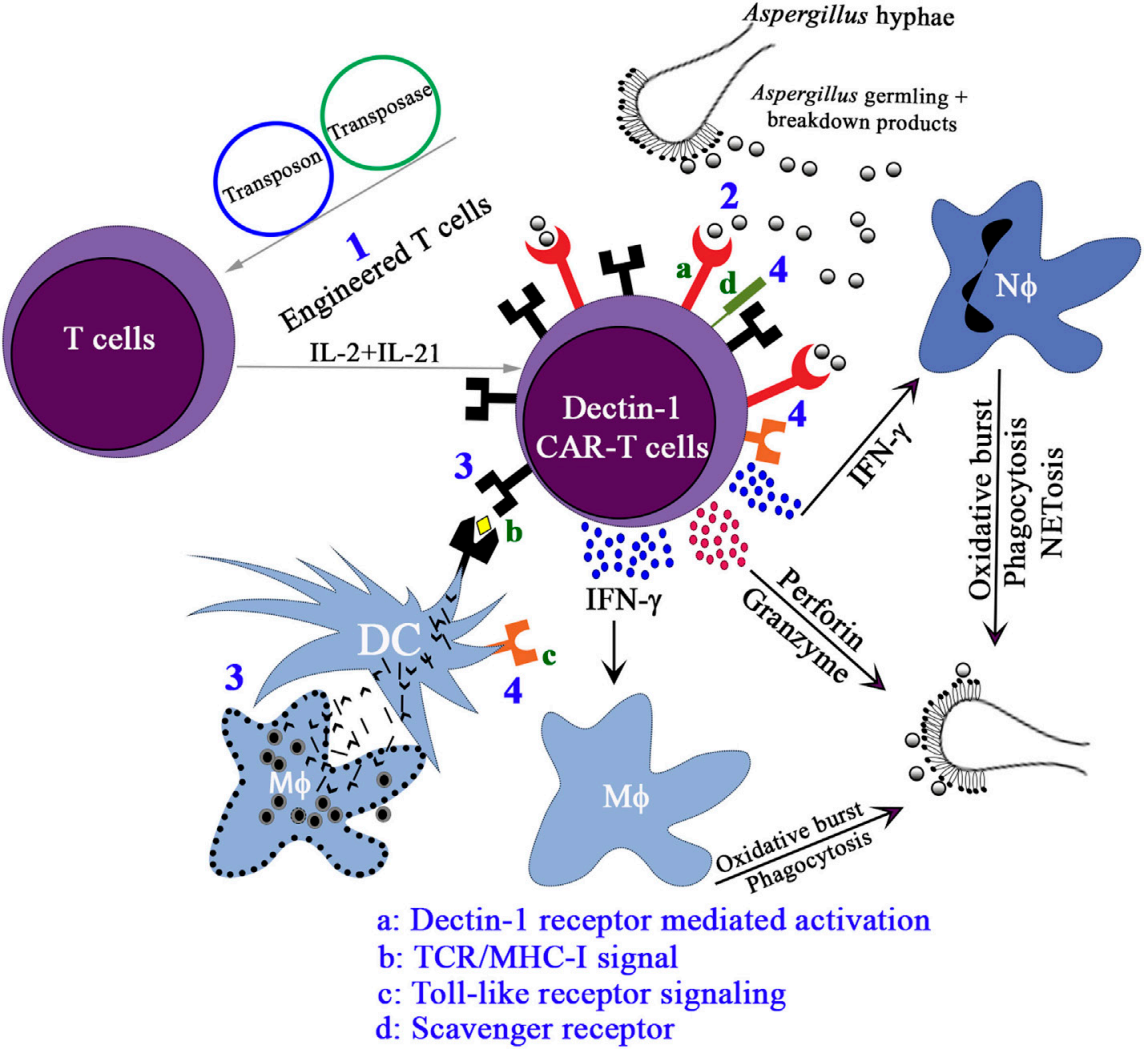
The proposed activation pathways of Engineered T cells. (1) Expression of D-CAR^+^ using Sleeping Beauty system, namely, D-CAR^+^ T cells; (2) the β-glucan expressing *Aspergillus* germlings are recognized by D-CAR^+^-T cells and induce the production of interferon (IFN)-γ, which favors the microbicidal activity of macrophages and neutrophils. Activated D-CAR^+^-T cells also secrete granzyme and perforin to degrade fungal cell walls. (3) The activation of the D-CAR^+^-T cells can also occur by cross-presentation of dendritic cells (DCs) and recognition by specific T-cell receptor (TCR), and (4) direct interaction of fungal breakdown products with toll-like receptors and scavenger receptor-ligands.

Since this was the first time that a PRR was adapted to redirect T-cell specificity, we employed multiple assays [cell viability assay (XTT), cytokine production, upregulation of CD107a, and microscopy] to compare the ability of D-CAR^+^ T cells to target germinating *Aspergillus* hyphae with that of CD19-specific T cells. All of these assays demonstrated that the D-CAR activated the cytolytic machinery of the genetically modified T cells and probably their perforin/granzyme pathway as well (126). The production of IFN-γ by the D-CAR^+^ T cells may further augment innate immunity to IFIs. Treatment with recombinant IFN-γ or IFN-γ derived from CD4^+^ helper T cells or NK cells has been shown to augment anti-*Aspergillus* activity (55, 122). It remains to be determined whether the D-CAR-dependent production of IFN-γ contributes directly to the clearance of fungal infections or whether this activity works indirectly through the activation of granulocytes. Other cytokines, such as IL-17, may also participate in antifungal immunity. Reports indicate that IL-17 can activate neutrophils in a similar manner to IFN-γ (158), though increased levels of IL-17 are associated with mortality (153, 159–162) (Figure 3).

The use of combination therapies may supplement the antifungal efficacy of D-CAR^+^ T cells. For example, in *Aspergillus* pre-exposed to Caspofungin, β-glucan residues in the cell wall were unmasked, enhancing antifungal activity mediated by neutrophils (163). In HSCT patients, innate immune cells are present in the blood within 2 weeks of the stem-cell transplant, whereas it takes, on average, 7–12 months for NK, B, and T cells to be produced. Most IFIs occur during this period because no cellular immune system exists to support the innate immune system (7, 164). One clinical application for the add-back of donor-derived D-CAR^+^-T cells after allogeneic HSCT is to provide protection from IFIs by the recognition of β-glucan moieties present in all opportunistic fungi.

### Bioengineered Dual CAR T Cells to Target B-Cell Leukemia and IFI

CD19-specific CAR T-cells have been used successfully to treat acute lymphoblastic leukemia (ALL) by eliminating both malignant and normal B-cells, since CD19 is also expressed on normal cells. However, total elimination of B-cells resulted in B-cell aplasia as a side effect (165). Therefore, patients undergoing CAR T cell therapy are typically given intravenous Ig to control bacterial and fungal infections. Also, high incidences of IFI are found in patients diagnosed with pediatric ALL (166, 167). These patients will gain additional benefits if CAR T cells can be engineered to destroy both IFI and tumor cells. With this goal in mind, we developed a novel gene therapy approach using dual CAR T cells to prevent IFIs such as *Aspergillus* and *Candida* and also treat B-cell leukemia. To target fungal infections, we adapted the PRR Dectin-1 to activate T cells *via* chimeric CD28 and CD3-ζ upon binding with β-1,3-gucan carbohydrate present in the fungal cell wall. The D-CAR^+^ T cells exhibited specificity for β-1,3-gucan and led to damage to fungal hyphae and inhibition of hyphal growth of *Aspergillus* and *Candida* upon testing in both *in vitro* and mouse models. To target B-cell leukemia, we adapted chimeric CD19R-CD28-CD3-ζ T-cells that are currently being used in clinical trials (149). The D-CAR^+^ T cells do not kill the yeast form of *Candida* so there should not be any reactions to normal commensals that live in the gut microbiota. Also, D-CAR T cells can control *Aspergillus* infections in the presence of immunosuppressive drugs at physiological concentrations (168). Thus, we propose utilizing the clinically appealing dual CAR T cells to control both leukemia and IFIs.

### Future Directions for CAR T-Cell Therapy

The recent breakthroughs in bioengineered CAR T-cell therapy for cancer have opened up new horizons for targeting infectious disease-causing organisms, such as viruses, bacteria, and fungi. This approach promises to be especially useful in immunocompromised patients or those requiring long-term immunosuppressive drug therapy, such as solid organ transplant recipients. CAR T-cell therapy offers not only an immediate cure of the disease, but also long-term benefits because memory CAR T cells will protect the host from future attack by foreign invaders. This therapy will also give new hope to patients suffering from drug-resistant IFIs such as aspergillosis. An advantage of D-CAR T cell therapy is that it can be used with antifungal therapy such as Caspofungin and Amphotericin-B, thereby reducing drug-related toxicity such as nephrotoxicity associated with Amphotericin-B.

Several factors limit the immediate clinical applications of CAR T-cell therapy. Cytokine storm and neurotoxicity are the major side effects of CAR T-cell therapy and the good news is that now clinicians are successfully addressing these symptoms (169). Since D-CAR^+^-T cells are activated by the β-glucan sugar moiety which is not present in the mammalian system, off-target related toxicities may be minimal. At present, we cannot rule out the possibility of other toxicities such as macrophage activation syndrome or GvHD that are observed in CAR T therapy to treat cancers (170).

At present, CAR T-cell therapy is a personalized therapy; more CAR T-cell manufacturing centers are needed to produce clinical-grade T cells in a cost-effective way. The therapeutic success of any form of ACT depends on infusing sufficient numbers of T cells that lack replicative senescence and terminal differentiation and have the desired specificity (171). The CAR T-cell therapy used in current clinical trials requires the use of a Good Manufacturing Practice (GMP)-compliant facility to generate the T cells; it takes 2–4 weeks to propagate enough CAR T cells for infusion into the patient. However, the length of time T cells spend in culture, especially if they are propagated under non-physiological conditions, may erode the quality of the product despite increasing its quantity. Thus, a technique to generate T cells that can be harvested from peripheral blood, minimally manipulated, and infused within a few days of collection is appealing. Pharmaceutical and biotechnology companies are actively evaluating methods for generating CAR T cells in less than a week by automating the cell culture process. Automation has immediate appeal, as it avoids the expense and risk of contamination associated with prolonged culture and reduces human labor-associated error. Rapid production may in fact improve the therapeutic potential of the manufactured T cells by allowing them to avoid the replication senescence and terminal differentiation that causes them to lose *in vivo* persistence.

Approaches to generating T cells in compliance with GMPs are based on the *ex vivo* use of reagents to identify antigen-specific T cells. One approach is to use fluorescence-labeled or paramagnetic-labeled probes that bind TCRs to identify T cells with the desired specificity. The labeled T cells are subsequently subjected to fluorescence-activated cell sorting or magnetic selection to generate a homogeneously tagged product that can be immediately infused upon meeting release criteria (172, 173). The success of this approach is measured in terms of the time needed to identify antigen-specific T cells and the specificity of the harvested product. In another approach, antigen-specific T cells are isolated from donor PBMCs using a cytokine-capture system. In this process, donor PBMCs are incubated with a peptide antigen for 4 h; the activated T cells secrete IFN-γ, which is captured by a magnetic bead-conjugated bi-specific antibody. One arm of the bi-specific antibody is specific to IFN-γ, and the other arm is specific for the cell-surface CD45. T cells that secrete IFN-γ are then separated by passing them through a column. However, this approach is limited by the number of antigen-specific T cells in the donor. If more than one donor is available, prescreening of T cells (obtained from potential donors by simple venipuncture) for antigen-specific secretion of IFN-γ will determine the most suitable donor.

Despite these limitations, adoptive transfer of viral-antigen-specific T cells that have been modified for minimal manipulation and immediate infusion has been successful in clinical trials (174). TCR sequences can be identified from these antigen-specific T cells and can be used to generate TCR CAR T cells (175). Some clinical applications, such as infusion of allogeneic antigen-specific T cells after HSCT, are not possible because the initial donor may be unavailable or anonymous. In these cases, however, potential recipients may benefit from infusion of “captured” T cells from third-party donors that can recognize antigens *via* a human leukocyte antigen molecule shared by the recipient and the donor. These “off-the-shelf” T cells could be premanufactured and cryopreserved for infusion on demand. This approach might be better for prophylaxis in high-risk patients than for treatment in patients with recalcitrant IFIs. A precedent for this approach was reported, in which third-party Epstein–Barr virus-specific T cells and multivirus-specific T cells were infused (119, 176, 177). This approach could be adapted to treat or prevent IFIs.

## Conclusion

Adoptive T-cell therapy could play a key role in controlling IFI. The GMP grade protocols for isolation of fungal-specific T cells are well characterized. Fungal-specific CD8^+^ T-cells protect the host by activating the host innate immune system (Tc1 mediated) and mucosal immune system (Tc17 mediated) against IFI. For direct control, D-CAR^+^ T-cells have been developed by fusing the extra cellular domain of Dectin-1 and cytoplasmic domains of CD3 and CD28 receptors. It can target various fungi, such as *Aspergillus* and *Candida* (126), and such treatment is highly warranted to combat IFI infections in immunocompromised patients. We have also developed Bi-specific CARs to target both B-cell malignancies and IFI by expressing CD19R-CAR and D-CAR in the same T-cell. The high costs involved with providing CAR T-cell therapy may prohibit many patients from receiving this potentially life-saving therapy, especially those located in the developing world, where fungal infections are highly prevalent. To reduce the manufacturing costs, off-the-shelf products are being developed which can be adapted for treating IFI in near future.

## Author Contributions

DPK wrote the introduction and back ground. TAS wrote CD8^+^ T cell vaccines and PRK wrote engineered T-cells and Dectin1-CAR T cells. All authors have equally contributed for the tables. TAS and PRK contributed equally for figures.

## Conflict of Interest Statement

Some of the technology described was advanced to clinic through research conducted at MD Anderson Cancer Center by PK and DK. A patent application based on research reported in this manuscript has been filed.
